# 3-Hy­droxy-1-methyl-2-[4-(piperidin-1-yl)phen­yl]quinolin-4(1*H*)-one

**DOI:** 10.1107/S1600536811005046

**Published:** 2011-02-19

**Authors:** Michał Wera, Vasyl G. Pivovarenko, Artur Sikorski, Tadeusz Lis, Jerzy Błażejowski

**Affiliations:** a Faculty of Chemistry, University of Gdańsk, J. Sobieskiego 18, 80-952 Gdańsk, Poland; b Faculty of Chemistry, Kyiv Taras Shevchenko National University, Volodymyrska 64, 01033 Kyiv, Ukraine; c Faculty of Chemistry, University of Wrocław, F. Joliot-Curie 14, 50-383 Wrocław, Poland

## Abstract

There are two structurally similar but crystallographically independent mol­ecules (*A* and *B*) in the asymmetric unit of the title compound, C_21_H_22_N_2_O_2_, which are linked *via* two O—H⋯O hydrogen bonds. An intramolecular O—H⋯O hydrogen bond also occurs in each molecule. In the crystal, the *A* and *B* mol­ecules are further linked through C—H⋯O inter­actions. The benzene ring is twisted at an angle of 69.9 (1) and 83.4 (1)° relative to the 1,4-dihydro­quinoline skeleton in mol­ecules *A* and *B*, respectively. Adjacent 1,4-dihydro­quinoline units of mol­ecules *A* are parallel, while mol­ecules *A* and *B* are oriented at an angle of 32.8 (1)°.

## Related literature

For general background to quinolin-4(1*H*)-ones, see: Bilokin’ *et al.* (2009) ▶; Mitscher (2005 ▶); Yushchenko *et al.* (2007 ▶); Sengupta & Kasha (1979 ▶). For related structures, see: Czaun *et al.* (2002 ▶); Mphahlele *et al.* (2002 ▶); Mphahlele & El-Nahas (2004 ▶). For inter­molecular inter­actions, see: Aakeröy *et al.* (1992 ▶); Novoa *et al.* (2006 ▶). For the synthesis, see: Hradil *et al.* (1999) ▶; Yushchenko *et al.* (2006 ▶).
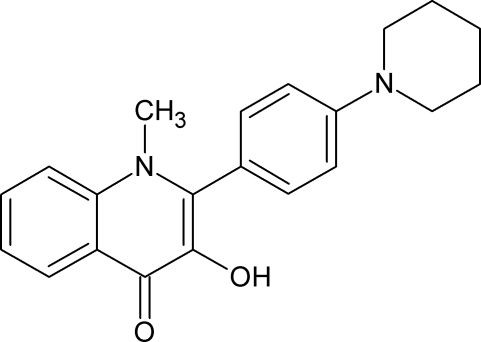


## Experimental

### 

#### Crystal data

C_21_H_22_N_2_O_2_
*M* *_r_* = 334.41
Monoclinic, 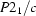

*a* = 9.621 (4) Å
*b* = 18.622 (6) Å
*c* = 18.955 (7) Å
β = 104.17 (3)°
*V* = 3293 (2) Å^3^
*Z* = 8
Mo *K*α radiation
μ = 0.09 mm^−1^
*T* = 180 K
0.40 × 0.35 × 0.30 mm

#### Data collection

Oxford Diffraction Xcalibur PX diffractometer with a CCD area detector
41668 measured reflections
13682 independent reflections
6901 reflections with *I* > 2σ(*I*)
*R*_int_ = 0.040

#### Refinement

*R*[*F* ^2^ > 2σ(*F* ^2^)] = 0.059
*wR*(*F* ^2^) = 0.133
*S* = 1.01
13682 reflections
455 parameters
H-atom parameters constrained
Δρ_max_ = 0.50 e Å^−3^
Δρ_min_ = −0.25 e Å^−3^

### 

Data collection: *CrysAlis CCD* (Oxford Diffraction, 2003 ▶); cell refinement: *CrysAlis RED* (Oxford Diffraction, 2003 ▶); data reduction: *CrysAlis RED*; program(s) used to solve structure: *SHELXS97* (Sheldrick, 2008 ▶); program(s) used to refine structure: *SHELXL97* (Sheldrick, 2008 ▶); molecular graphics: *ORTEP-3* (Farrugia, 1997 ▶); software used to prepare material for publication: *SHELXL97* and *PLATON* (Spek, 2009 ▶).

## Supplementary Material

Crystal structure: contains datablocks global, I. DOI: 10.1107/S1600536811005046/om2406sup1.cif

Structure factors: contains datablocks I. DOI: 10.1107/S1600536811005046/om2406Isup2.hkl

Additional supplementary materials:  crystallographic information; 3D view; checkCIF report

## Figures and Tables

**Table 1:** Hydrogen-bond geometry (Å, °)

*D*—H⋯*A*	*D*—H	H⋯*A*	*D*⋯*A*	*D*—H⋯*A*
O11*A*—H11*A*⋯O12*A*	0.84	2.32	2.760 (2)	113
O11*A*—H11*A*⋯O12*B*	0.84	1.95	2.723 (2)	153
O11*B*—H11*B*⋯O12*A*	0.84	2.01	2.791 (2)	154
O11*B*—H11*B*⋯O12*B*	0.84	2.30	2.741 (2)	113
C24*B*—H24*D*⋯O12*A*^i^	0.99	2.52	3.475 (2)	162

Symmetry code: (i) 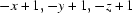.

**Fig. 1.**
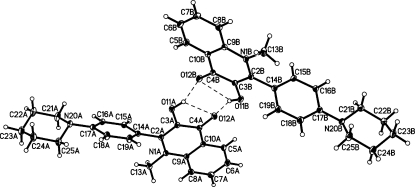
The structure of molecules A and B of the title compound together with the atom labeling scheme. Displacement ellipsoids are drawn at the 25% probability level, and H atoms are shown as small spheres of arbitrary radius. The O–H···O hydrogen bonds are represented by dashed lines.

**Fig. 2.**
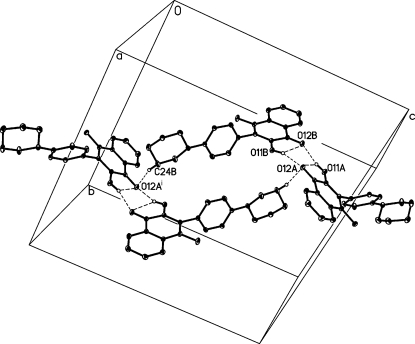
The arrangement of molecules A and B in the crystal. The O–H···O interactions are represented by dashed lines, the C–H···O contacts by dotted lines. H atoms not involved in interactions have been omitted. [Symmetry code: (i) –x + 1, –y + 1, –z + 1.]

## supplementary crystallographic information

### Comment

Apart from their interesting biological activities (Mitscher, 2005), quinolin-4(1*H*)-ones display dual fluorescence, the result of Excited State Intramolecular Proton Transfer (ESIPT), if they are substituted with –OH and phenyl in the pyridine-4(1*H*)-one ring in the vicinity of the carbonyl group and the N atom, respectively (Yushchenko *et al.*, 2007). Influenced by the properties of the medium, ESIPT makes 3-hydroxy-2-phenylquinolin-4(1*H*)-ones interesting fluorescent probes sensitive to features of a medium (Bilokin' *et al.*, 2009). Since ESIPT is believed to depend on the mutual orientation of the 1,4-dihydroquinoline and benzene fragments (Yushchenko *et al.*, 2007), we undertook investigations into the structure of potential fluorescent sensors belonging to the latter group of compounds. Here the structure of 3-hydroxy-1-methyl-(-2-[4-(piperidin-1-yl)phenyl]quinolin-4(1*H*)-one is presented.

In the title compound (Fig. 1), the bond lengths and angles characterizing the geometry of the 2-phenylquinolin-4(1*H*)-one moiety are typical of this group of compounds (Czaun *et al.*, 2002; Mphahlele *et al.*, 2002; Mphahlele & El-Nahas, 2004). With respective average deviations from planarity of 0.0163 (1)° (A) or 0.0180 (1)° (B) and 0.0078 (1)° (A) or 0.0059 (1)° (B), the 1,4-dihydroquinoline and benzene ring systems are oriented at a dihedral angle of 69.9 (1)° (A) or 83.4 (1)° (B) (in crystalline 3-hydroxy-2-phenylquinolin-4(1*H*)-one: dimethyl sulfoxide, 1:1, this angle is equal to 45.2 (1)° (Czaun *et al.*, 2002)). As mentioned above, the latter angle appears to be important for explaining the mechanism of ESIPT in this group of compounds (Yushchenko *et al.*, 2007).

In the crystal lattice, two structurally similar but crystallographically independent molecules (A and B), linked *via* two O–H···O hydrogen bonds (Aakeröy *et al.*, 1992), are present in the asymmetric unit (Table 1, Fig. 1). Molecules A are in contact with neighboring B ones through C–H···O (Novoa *et al.*, 2006) interactions (Table 1, Fig. 2). Adjacent 1,4-dihydroquinoline units of molecules A are parallel – they lie at an angle of 0.0 (1)° – while molecules A and B are oriented at an angle of 32.8 (1)°. The O12–H12···O13 intramolecular hydrogen bonds (Table 1, Figs. 1 and 2) are the ones that may be involved in ESIPT; the phenomenon originally disclosed in 3-hydroxy-2-phenyl-4*H*-chromen-4-ones (Sengupta & Kasha, 1979), which are analogues of 3-hydroxy-2-phenylquinolin-4(1*H*)-ones.

### Experimental

The title compound was synthesized in two steps. First a mixture of 2-(methylamino)benzoic acid, 2-bromo-1-(4-fluorophenyl)ethanone and potassium carbonate in dimethylformamide was heated at 325 K for 1 h to obtain 2-(4-fluorophenyl)-2-oxoethyl 2-(methylamino)benzoate. On further heating with polyphosphoric acid (395 K, 2 h), this yielded 2-(4-fluorophenyl)-3-hydroxy-1-methyl-quinolin-4(1*H*)-one (Hradil *et al.*, 1999; Yushchenko *et al.*, 2006). The latter compound was then separated, dissolved in piperidine and the solution stored in a sealed tube at 445 K for 50 h. The reactant mixture was subsequently poured into 1% aq HCl and the precipitate separated by filtration. Crystals suitable for X-ray investigations were grown from dimethylformamide (m.p. = 566–568 K).

The X-ray measurements were carried out at 180 K. Below this temperature, a phase transition occurs, which doubles parameter *c* of the unit cell.

### Refinement

H atoms of C–H bonds were positioned geometrically, with C–H = 0.95 Å, 0.98 Å and 0.99 Å for the aromatic, methyl and methylene H atoms, respectively, and constrained to ride on their parent atoms with *U*_iso_(H) = *xU*_eq_(C), where *x* = 1.2 for the aromatic and 1.5 for alkyl H atoms. H atoms of O–H bonds were positioned geometrically with O–H = 0.84 Å, and constrained to ride on their parent atoms with *U*_iso_(H) = 1.5*U*_eq_(O).

### Crystal data

**Table:**

C_21_H_22_N_2_O_2_	*F*(000) = 1424
*M**_r_* = 334.41	*D*_x_ = 1.349 Mg m^−^^3^
Monoclinic, *P*2_1_/*c*	Mo *K*α radiation, λ = 0.71073 Å
Hall symbol: -P 2ybc	Cell parameters from 19567 reflections
*a* = 9.621 (4) Å	θ = 3–35°
*b* = 18.622 (6) Å	µ = 0.09 mm^−^^1^
*c* = 18.955 (7) Å	*T* = 180 K
β = 104.17 (3)°	Block, colorless
*V* = 3293 (2) Å^3^	0.40 × 0.35 × 0.30 mm
*Z* = 8	

### Data collection

**Table:**

Oxford Diffraction Xcalibur PX diffractometer with a CCD area detector'	6901 reflections with *I* > 2σ(*I*)
Radiation source: fine-focus sealed tube	*R*_int_ = 0.040
graphite	θ_max_ = 35.1°, θ_min_ = 3.1°
ω and φ scans	*h* = −15→13
41668 measured reflections	*k* = −30→24
13682 independent reflections	*l* = −30→30

### Refinement

**Table:**

Refinement on *F*^2^	Primary atom site location: structure-invariant direct methods
Least-squares matrix: full	Secondary atom site location: difference Fourier map
*R*[*F*^2^ > 2σ(*F*^2^)] = 0.059	Hydrogen site location: inferred from neighbouring sites
*wR*(*F*^2^) = 0.133	H-atom parameters constrained
*S* = 1.01	*w* = 1/[σ^2^(*F*_o_^2^) + (0.053*P*)^2^] where *P* = (*F*_o_^2^ + 2*F*_c_^2^)/3
13682 reflections	(Δ/σ)_max_ = 0.001
455 parameters	Δρ_max_ = 0.50 e Å^−^^3^
0 restraints	Δρ_min_ = −0.25 e Å^−^^3^

### Special details

**Table:**

Geometry. All e.s.d.'s (except the e.s.d. in the dihedral angle between two l.s. planes) are estimated using the full covariance matrix. The cell e.s.d.'s are taken into account individually in the estimation of e.s.d.'s in distances, angles and torsion angles; correlations between e.s.d.'s in cell parameters are only used when they are defined by crystal symmetry. An approximate (isotropic) treatment of cell e.s.d.'s is used for estimating e.s.d.'s involving l.s. planes.
Refinement. Refinement of *F*^2^ against ALL reflections. The weighted *R*-factor *wR* and goodness of fit *S* are based on *F*^2^, conventional *R*-factors *R* are based on *F*, with *F* set to zero for negative *F*^2^. The threshold expression of *F*^2^ > σ(*F*^2^) is used only for calculating *R*-factors(gt) *etc*. and is not relevant to the choice of reflections for refinement. *R*-factors based on *F*^2^ are statistically about twice as large as those based on *F*, and *R*- factors based on ALL data will be even larger.

### Fractional atomic coordinates and isotropic or equivalent isotropic displacement parameters (Å^2^)

**Table:**

	*x*	*y*	*z*	*U*_iso_*/*U*_eq_	
N1A	0.62758 (10)	0.35031 (6)	0.98211 (5)	0.0217 (2)	
N1B	−0.01996 (10)	0.36909 (6)	0.49772 (5)	0.0227 (2)	
C2A	0.48185 (12)	0.35224 (6)	0.95022 (6)	0.0202 (2)	
C2B	0.12649 (12)	0.36293 (6)	0.52659 (6)	0.0204 (2)	
C3A	0.42999 (12)	0.33671 (6)	0.87742 (6)	0.0202 (2)	
C3B	0.17973 (12)	0.34211 (6)	0.59775 (6)	0.0203 (2)	
C4A	0.52258 (12)	0.31590 (6)	0.83202 (6)	0.0193 (2)	
C4B	0.08833 (13)	0.32634 (7)	0.64502 (6)	0.0219 (2)	
C5A	0.77324 (13)	0.29184 (7)	0.82809 (7)	0.0251 (3)	
H5A	0.7395	0.2782	0.7785	0.030*	
C5B	−0.16279 (13)	0.31646 (8)	0.65442 (7)	0.0286 (3)	
H5B	−0.1281	0.3033	0.7040	0.034*	
C6A	0.91803 (14)	0.29110 (7)	0.85969 (7)	0.0301 (3)	
H6A	0.9841	0.2778	0.8321	0.036*	
C6B	−0.30772 (14)	0.32021 (8)	0.62521 (7)	0.0318 (3)	
H6B	−0.3729	0.3102	0.6544	0.038*	
C7A	0.96681 (14)	0.31024 (8)	0.93299 (7)	0.0299 (3)	
H7A	1.0669	0.3104	0.9546	0.036*	
C7B	−0.35874 (13)	0.33903 (7)	0.55167 (7)	0.0283 (3)	
H7B	−0.4592	0.3410	0.5311	0.034*	
C8A	0.87355 (13)	0.32885 (7)	0.97444 (7)	0.0264 (3)	
H8A	0.9089	0.3405	1.0244	0.032*	
C8B	−0.26632 (13)	0.35457 (7)	0.50900 (7)	0.0259 (3)	
H8B	−0.3030	0.3670	0.4593	0.031*	
C9A	0.72446 (13)	0.33055 (6)	0.94225 (6)	0.0203 (2)	
C9B	−0.11638 (12)	0.35211 (6)	0.53882 (6)	0.0204 (2)	
C10A	0.67400 (12)	0.31256 (6)	0.86814 (6)	0.0197 (2)	
C10B	−0.06406 (12)	0.33181 (7)	0.61233 (6)	0.0214 (2)	
O11A	0.28603 (9)	0.33866 (5)	0.84855 (5)	0.0281 (2)	
H11A	0.2683	0.3277	0.8042	0.042*	
O11B	0.32439 (9)	0.33503 (5)	0.62420 (5)	0.0257 (2)	
H11B	0.3421	0.3211	0.6676	0.039*	
O12A	0.47359 (9)	0.30034 (5)	0.76573 (4)	0.0259 (2)	
O12B	0.13899 (9)	0.30750 (6)	0.71000 (5)	0.0320 (2)	
C13A	0.68432 (14)	0.37058 (8)	1.05886 (6)	0.0283 (3)	
H13A	0.6080	0.3929	1.0774	0.042*	
H13B	0.7633	0.4048	1.0627	0.042*	
H13C	0.7194	0.3276	1.0876	0.042*	
C13B	−0.07655 (14)	0.39398 (8)	0.42239 (7)	0.0325 (3)	
H13D	−0.1427	0.4341	0.4220	0.049*	
H13E	−0.1276	0.3545	0.3928	0.049*	
H13F	0.0030	0.4099	0.4023	0.049*	
C14A	0.38208 (13)	0.37134 (7)	0.99616 (6)	0.0211 (2)	
C14B	0.22385 (12)	0.37982 (7)	0.47866 (6)	0.0212 (2)	
C15A	0.35906 (13)	0.32537 (7)	1.04993 (6)	0.0232 (3)	
H15A	0.4066	0.2803	1.0570	0.028*	
C15B	0.25605 (13)	0.32892 (7)	0.43131 (7)	0.0239 (3)	
H15B	0.2171	0.2820	0.4304	0.029*	
C16A	0.26762 (13)	0.34414 (7)	1.09372 (6)	0.0232 (3)	
H16A	0.2544	0.3117	1.1302	0.028*	
C16B	0.34430 (13)	0.34540 (7)	0.38519 (7)	0.0235 (3)	
H16B	0.3639	0.3096	0.3533	0.028*	
C17A	0.19498 (13)	0.40993 (7)	1.08483 (6)	0.0232 (3)	
C17B	0.40451 (12)	0.41371 (6)	0.38511 (6)	0.0206 (2)	
C18A	0.21631 (15)	0.45534 (7)	1.02923 (7)	0.0312 (3)	
H18A	0.1676	0.5001	1.0211	0.037*	
C18B	0.36954 (15)	0.46512 (7)	0.43209 (7)	0.0318 (3)	
H18B	0.4059	0.5126	0.4324	0.038*	
C19A	0.30755 (15)	0.43583 (7)	0.98596 (7)	0.0306 (3)	
H19A	0.3192	0.4674	0.9485	0.037*	
C19B	0.28269 (15)	0.44759 (7)	0.47807 (7)	0.0315 (3)	
H19B	0.2629	0.4832	0.5102	0.038*	
N20A	0.10083 (11)	0.42989 (6)	1.12809 (5)	0.0251 (2)	
N20B	0.49979 (11)	0.43049 (5)	0.34169 (5)	0.0228 (2)	
C21A	0.09634 (16)	0.38008 (9)	1.18785 (8)	0.0382 (4)	
H21A	0.1923	0.3784	1.2220	0.046*	
H21B	0.0739	0.3313	1.1676	0.046*	
C21B	0.51323 (15)	0.37533 (7)	0.28849 (7)	0.0312 (3)	
H21C	0.4189	0.3690	0.2536	0.037*	
H21D	0.5389	0.3292	0.3143	0.037*	
C22A	−0.01350 (16)	0.40103 (9)	1.22943 (8)	0.0387 (4)	
H22A	−0.1109	0.3954	1.1974	0.046*	
H22B	−0.0051	0.3683	1.2714	0.046*	
C22B	0.62434 (14)	0.39258 (8)	0.24620 (7)	0.0304 (3)	
H22C	0.7214	0.3891	0.2791	0.036*	
H22D	0.6177	0.3568	0.2069	0.036*	
C23A	0.00603 (16)	0.47724 (9)	1.25640 (8)	0.0413 (4)	
H23A	0.0999	0.4825	1.2919	0.050*	
H23B	−0.0703	0.4900	1.2810	0.050*	
C23B	0.60333 (15)	0.46696 (8)	0.21334 (7)	0.0329 (3)	
H23C	0.5094	0.4701	0.1776	0.039*	
H23D	0.6795	0.4776	0.1880	0.039*	
C24A	−0.00105 (18)	0.52637 (8)	1.19200 (9)	0.0441 (4)	
H24A	0.0162	0.5765	1.2094	0.053*	
H24B	−0.0983	0.5240	1.1591	0.053*	
C24B	0.60962 (16)	0.52036 (7)	0.27452 (8)	0.0352 (3)	
H24C	0.7057	0.5183	0.3086	0.042*	
H24D	0.5956	0.5695	0.2540	0.042*	
C25A	0.10870 (17)	0.50598 (8)	1.14988 (9)	0.0414 (4)	
H25A	0.0936	0.5363	1.1057	0.050*	
H25B	0.2059	0.5162	1.1803	0.050*	
C25B	0.49643 (16)	0.50524 (7)	0.31595 (8)	0.0336 (3)	
H25C	0.5113	0.5380	0.3583	0.040*	
H25D	0.4006	0.5155	0.2840	0.040*	

### Atomic displacement parameters (Å^2^)

**Table:**

	*U*^11^	*U*^22^	*U*^33^	*U*^12^	*U*^13^	*U*^23^
N1A	0.0208 (5)	0.0283 (6)	0.0166 (5)	0.0005 (4)	0.0060 (4)	−0.0027 (4)
N1B	0.0199 (5)	0.0330 (6)	0.0158 (5)	0.0022 (4)	0.0056 (4)	0.0024 (4)
C2A	0.0211 (6)	0.0213 (6)	0.0205 (6)	0.0020 (5)	0.0094 (5)	0.0009 (5)
C2B	0.0202 (6)	0.0238 (6)	0.0199 (5)	0.0001 (5)	0.0098 (5)	−0.0005 (5)
C3A	0.0177 (6)	0.0248 (6)	0.0191 (5)	0.0024 (5)	0.0067 (5)	0.0012 (5)
C3B	0.0171 (6)	0.0256 (6)	0.0189 (5)	−0.0007 (5)	0.0059 (5)	−0.0009 (5)
C4A	0.0209 (6)	0.0198 (6)	0.0186 (5)	0.0007 (5)	0.0073 (5)	0.0014 (5)
C4B	0.0195 (6)	0.0293 (7)	0.0175 (5)	−0.0022 (5)	0.0060 (5)	0.0001 (5)
C5A	0.0233 (6)	0.0334 (7)	0.0208 (6)	0.0018 (5)	0.0093 (5)	−0.0015 (5)
C5B	0.0218 (6)	0.0441 (8)	0.0216 (6)	−0.0016 (6)	0.0085 (5)	0.0046 (6)
C6A	0.0220 (6)	0.0402 (8)	0.0309 (7)	0.0038 (6)	0.0119 (6)	−0.0022 (6)
C6B	0.0219 (6)	0.0437 (8)	0.0328 (7)	−0.0025 (6)	0.0126 (6)	0.0034 (6)
C7A	0.0190 (6)	0.0365 (8)	0.0334 (7)	0.0031 (5)	0.0048 (6)	−0.0035 (6)
C7B	0.0166 (6)	0.0346 (8)	0.0330 (7)	0.0000 (5)	0.0047 (5)	0.0026 (6)
C8A	0.0220 (6)	0.0326 (7)	0.0234 (6)	0.0014 (5)	0.0033 (5)	−0.0029 (5)
C8B	0.0214 (6)	0.0319 (7)	0.0227 (6)	0.0019 (5)	0.0025 (5)	0.0016 (5)
C9A	0.0214 (6)	0.0224 (6)	0.0184 (5)	0.0002 (5)	0.0071 (5)	−0.0003 (5)
C9B	0.0193 (6)	0.0242 (6)	0.0187 (5)	0.0000 (5)	0.0067 (5)	−0.0011 (5)
C10A	0.0193 (6)	0.0225 (6)	0.0187 (5)	0.0008 (5)	0.0072 (5)	0.0012 (5)
C10B	0.0189 (6)	0.0281 (7)	0.0181 (5)	−0.0021 (5)	0.0063 (5)	0.0000 (5)
O11A	0.0186 (4)	0.0456 (6)	0.0204 (4)	0.0042 (4)	0.0055 (4)	−0.0026 (4)
O11B	0.0173 (4)	0.0390 (6)	0.0214 (4)	−0.0008 (4)	0.0059 (3)	0.0030 (4)
O12A	0.0242 (5)	0.0354 (5)	0.0178 (4)	0.0042 (4)	0.0044 (4)	−0.0007 (4)
O12B	0.0226 (5)	0.0537 (6)	0.0193 (4)	−0.0044 (4)	0.0040 (4)	0.0058 (4)
C13A	0.0290 (7)	0.0360 (8)	0.0201 (6)	−0.0034 (6)	0.0066 (5)	−0.0054 (5)
C13B	0.0295 (7)	0.0499 (9)	0.0189 (6)	0.0089 (6)	0.0074 (5)	0.0091 (6)
C14A	0.0214 (6)	0.0252 (6)	0.0182 (5)	0.0007 (5)	0.0077 (5)	−0.0017 (5)
C14B	0.0191 (6)	0.0276 (7)	0.0181 (5)	0.0001 (5)	0.0070 (5)	0.0012 (5)
C15A	0.0226 (6)	0.0260 (6)	0.0227 (6)	0.0030 (5)	0.0089 (5)	0.0010 (5)
C15B	0.0242 (6)	0.0247 (6)	0.0257 (6)	−0.0031 (5)	0.0114 (5)	0.0006 (5)
C16A	0.0237 (6)	0.0276 (7)	0.0205 (6)	0.0013 (5)	0.0099 (5)	0.0023 (5)
C16B	0.0250 (6)	0.0244 (6)	0.0246 (6)	−0.0014 (5)	0.0127 (5)	−0.0023 (5)
C17A	0.0209 (6)	0.0299 (7)	0.0201 (6)	0.0012 (5)	0.0074 (5)	−0.0014 (5)
C17B	0.0199 (6)	0.0237 (6)	0.0200 (6)	0.0003 (5)	0.0082 (5)	0.0008 (5)
C18A	0.0374 (7)	0.0308 (7)	0.0307 (7)	0.0120 (6)	0.0186 (6)	0.0078 (6)
C18B	0.0441 (8)	0.0243 (7)	0.0340 (7)	−0.0074 (6)	0.0231 (6)	−0.0047 (6)
C19A	0.0388 (8)	0.0307 (7)	0.0280 (7)	0.0076 (6)	0.0192 (6)	0.0076 (6)
C19B	0.0432 (8)	0.0256 (7)	0.0333 (7)	−0.0040 (6)	0.0241 (6)	−0.0071 (6)
N20A	0.0258 (5)	0.0292 (6)	0.0238 (5)	0.0037 (5)	0.0131 (5)	0.0012 (4)
N20B	0.0259 (5)	0.0215 (5)	0.0247 (5)	−0.0015 (4)	0.0133 (4)	0.0003 (4)
C21A	0.0403 (8)	0.0473 (9)	0.0349 (8)	0.0146 (7)	0.0245 (7)	0.0140 (7)
C21B	0.0340 (7)	0.0312 (7)	0.0346 (7)	−0.0067 (6)	0.0203 (6)	−0.0081 (6)
C22A	0.0386 (8)	0.0528 (10)	0.0326 (7)	0.0115 (7)	0.0236 (7)	0.0100 (7)
C22B	0.0313 (7)	0.0359 (8)	0.0288 (7)	−0.0021 (6)	0.0169 (6)	−0.0038 (6)
C23A	0.0339 (8)	0.0656 (11)	0.0280 (7)	0.0033 (7)	0.0146 (6)	−0.0062 (7)
C23B	0.0339 (7)	0.0434 (8)	0.0243 (6)	0.0013 (6)	0.0128 (6)	0.0064 (6)
C24A	0.0555 (10)	0.0388 (9)	0.0498 (9)	0.0015 (7)	0.0353 (8)	−0.0071 (7)
C24B	0.0471 (8)	0.0283 (7)	0.0385 (8)	−0.0014 (6)	0.0260 (7)	0.0063 (6)
C25A	0.0518 (9)	0.0355 (8)	0.0478 (9)	−0.0040 (7)	0.0332 (8)	−0.0098 (7)
C25B	0.0428 (8)	0.0268 (7)	0.0382 (8)	0.0044 (6)	0.0236 (7)	0.0069 (6)

### Geometric parameters (Å, °)

**Table:**

N1A—C2A	1.3848 (16)	C15A—C16A	1.3938 (16)
N1A—C9A	1.3856 (15)	C15A—H15A	0.9500
N1A—C13A	1.4723 (16)	C15B—C16B	1.3938 (16)
N1B—C2B	1.3858 (16)	C15B—H15B	0.9500
N1B—C9B	1.3861 (15)	C16A—C17A	1.3999 (18)
N1B—C13B	1.4728 (16)	C16A—H16A	0.9500
C2A—C3A	1.3783 (17)	C16B—C17B	1.3979 (17)
C2A—C14A	1.4884 (16)	C16B—H16B	0.9500
C2B—C3B	1.3761 (17)	C17A—C18A	1.4048 (17)
C2B—C14B	1.4895 (16)	C17A—N20A	1.4124 (15)
C3A—O11A	1.3594 (15)	C17B—C18B	1.4035 (17)
C3A—C4A	1.4352 (16)	C17B—N20B	1.4088 (15)
C3B—O11B	1.3653 (15)	C18A—C19A	1.3885 (17)
C3B—C4B	1.4311 (16)	C18A—H18A	0.9500
C4A—O12A	1.2628 (14)	C18B—C19B	1.3861 (17)
C4A—C10A	1.4514 (18)	C18B—H18B	0.9500
C4B—O12B	1.2586 (15)	C19A—H19A	0.9500
C4B—C10B	1.4489 (18)	C19B—H19B	0.9500
C5A—C6A	1.3751 (19)	N20A—C25A	1.4727 (18)
C5A—C10A	1.4113 (16)	N20A—C21A	1.4729 (17)
C5A—H5A	0.9500	N20B—C21B	1.4670 (16)
C5B—C6B	1.3703 (19)	N20B—C25B	1.4727 (17)
C5B—C10B	1.4112 (16)	C21A—C22A	1.5159 (17)
C5B—H5B	0.9500	C21A—H21A	0.9900
C6A—C7A	1.3995 (19)	C21A—H21B	0.9900
C6A—H6A	0.9500	C21B—C22B	1.5192 (17)
C6B—C7B	1.4043 (19)	C21B—H21C	0.9900
C6B—H6B	0.9500	C21B—H21D	0.9900
C7A—C8A	1.3738 (18)	C22A—C23A	1.504 (2)
C7A—H7A	0.9500	C22A—H22A	0.9900
C7B—C8B	1.3713 (17)	C22A—H22B	0.9900
C7B—H7B	0.9500	C22B—C23B	1.512 (2)
C8A—C9A	1.4150 (18)	C22B—H22C	0.9900
C8A—H8A	0.9500	C22B—H22D	0.9900
C8B—C9B	1.4154 (18)	C23A—C24A	1.514 (2)
C8B—H8B	0.9500	C23A—H23A	0.9900
C9A—C10A	1.4097 (17)	C23A—H23B	0.9900
C9B—C10B	1.4123 (17)	C23B—C24B	1.517 (2)
O11A—H11A	0.8400	C23B—H23C	0.9900
O11B—H11B	0.8400	C23B—H23D	0.9900
C13A—H13A	0.9800	C24A—C25A	1.5196 (19)
C13A—H13B	0.9800	C24A—H24A	0.9900
C13A—H13C	0.9800	C24A—H24B	0.9900
C13B—H13D	0.9800	C24B—C25B	1.5167 (18)
C13B—H13E	0.9800	C24B—H24C	0.9900
C13B—H13F	0.9800	C24B—H24D	0.9900
C14A—C19A	1.3877 (18)	C25A—H25A	0.9900
C14A—C15A	1.3897 (17)	C25A—H25B	0.9900
C14B—C19B	1.3844 (18)	C25B—H25C	0.9900
C14B—C15B	1.3913 (17)	C25B—H25D	0.9900
			
C2A—N1A—C9A	120.87 (10)	C17A—C16A—H16A	119.4
C2A—N1A—C13A	121.04 (10)	C15B—C16B—C17B	121.14 (11)
C9A—N1A—C13A	118.08 (10)	C15B—C16B—H16B	119.4
C2B—N1B—C9B	120.98 (10)	C17B—C16B—H16B	119.4
C2B—N1B—C13B	120.51 (10)	C16A—C17A—C18A	117.18 (11)
C9B—N1B—C13B	118.50 (10)	C16A—C17A—N20A	122.18 (11)
C3A—C2A—N1A	120.75 (10)	C18A—C17A—N20A	120.62 (11)
C3A—C2A—C14A	120.57 (11)	C16B—C17B—C18B	117.15 (11)
N1A—C2A—C14A	118.67 (10)	C16B—C17B—N20B	122.12 (10)
C3B—C2B—N1B	120.59 (10)	C18B—C17B—N20B	120.71 (11)
C3B—C2B—C14B	121.24 (11)	C19A—C18A—C17A	121.01 (12)
N1B—C2B—C14B	118.17 (10)	C19A—C18A—H18A	119.5
O11A—C3A—C2A	118.74 (10)	C17A—C18A—H18A	119.5
O11A—C3A—C4A	119.12 (10)	C19B—C18B—C17B	120.89 (12)
C2A—C3A—C4A	122.10 (11)	C19B—C18B—H18B	119.6
O11B—C3B—C2B	119.24 (10)	C17B—C18B—H18B	119.6
O11B—C3B—C4B	118.53 (10)	C14A—C19A—C18A	121.60 (12)
C2B—C3B—C4B	122.22 (11)	C14A—C19A—H19A	119.2
O12A—C4A—C3A	121.55 (11)	C18A—C19A—H19A	119.2
O12A—C4A—C10A	123.13 (10)	C14B—C19B—C18B	122.04 (11)
C3A—C4A—C10A	115.30 (11)	C14B—C19B—H19B	119.0
O12B—C4B—C3B	121.33 (11)	C18B—C19B—H19B	119.0
O12B—C4B—C10B	123.16 (10)	C17A—N20A—C25A	114.83 (10)
C3B—C4B—C10B	115.49 (10)	C17A—N20A—C21A	115.13 (10)
C6A—C5A—C10A	121.00 (12)	C25A—N20A—C21A	113.53 (11)
C6A—C5A—H5A	119.5	C17B—N20B—C21B	115.19 (10)
C10A—C5A—H5A	119.5	C17B—N20B—C25B	116.01 (9)
C6B—C5B—C10B	121.36 (12)	C21B—N20B—C25B	115.63 (10)
C6B—C5B—H5B	119.3	N20A—C21A—C22A	113.16 (12)
C10B—C5B—H5B	119.3	N20A—C21A—H21A	108.9
C5A—C6A—C7A	119.19 (11)	C22A—C21A—H21A	108.9
C5A—C6A—H6A	120.4	N20A—C21A—H21B	108.9
C7A—C6A—H6A	120.4	C22A—C21A—H21B	108.9
C5B—C6B—C7B	119.19 (12)	H21A—C21A—H21B	107.8
C5B—C6B—H6B	120.4	N20B—C21B—C22B	113.98 (11)
C7B—C6B—H6B	120.4	N20B—C21B—H21C	108.8
C8A—C7A—C6A	121.65 (12)	C22B—C21B—H21C	108.8
C8A—C7A—H7A	119.2	N20B—C21B—H21D	108.8
C6A—C7A—H7A	119.2	C22B—C21B—H21D	108.8
C8B—C7B—C6B	121.23 (12)	H21C—C21B—H21D	107.7
C8B—C7B—H7B	119.4	C23A—C22A—C21A	111.99 (13)
C6B—C7B—H7B	119.4	C23A—C22A—H22A	109.2
C7A—C8A—C9A	119.61 (12)	C21A—C22A—H22A	109.2
C7A—C8A—H8A	120.2	C23A—C22A—H22B	109.2
C9A—C8A—H8A	120.2	C21A—C22A—H22B	109.2
C7B—C8B—C9B	120.16 (12)	H22A—C22A—H22B	107.9
C7B—C8B—H8B	119.9	C23B—C22B—C21B	111.76 (11)
C9B—C8B—H8B	119.9	C23B—C22B—H22C	109.3
N1A—C9A—C10A	119.54 (11)	C21B—C22B—H22C	109.3
N1A—C9A—C8A	121.13 (11)	C23B—C22B—H22D	109.3
C10A—C9A—C8A	119.33 (10)	C21B—C22B—H22D	109.3
N1B—C9B—C10B	119.32 (11)	H22C—C22B—H22D	107.9
N1B—C9B—C8B	121.68 (11)	C22A—C23A—C24A	108.59 (12)
C10B—C9B—C8B	119.00 (10)	C22A—C23A—H23A	110.0
C9A—C10A—C5A	119.19 (11)	C24A—C23A—H23A	110.0
C9A—C10A—C4A	121.40 (10)	C22A—C23A—H23B	110.0
C5A—C10A—C4A	119.41 (11)	C24A—C23A—H23B	110.0
C5B—C10B—C9B	119.03 (11)	H23A—C23A—H23B	108.4
C5B—C10B—C4B	119.65 (11)	C22B—C23B—C24B	108.01 (11)
C9B—C10B—C4B	121.31 (10)	C22B—C23B—H23C	110.1
C3A—O11A—H11A	109.5	C24B—C23B—H23C	110.1
C3B—O11B—H11B	109.5	C22B—C23B—H23D	110.1
N1A—C13A—H13A	109.5	C24B—C23B—H23D	110.1
N1A—C13A—H13B	109.5	H23C—C23B—H23D	108.4
H13A—C13A—H13B	109.5	C23A—C24A—C25A	111.89 (13)
N1A—C13A—H13C	109.5	C23A—C24A—H24A	109.2
H13A—C13A—H13C	109.5	C25A—C24A—H24A	109.2
H13B—C13A—H13C	109.5	C23A—C24A—H24B	109.2
N1B—C13B—H13D	109.5	C25A—C24A—H24B	109.2
N1B—C13B—H13E	109.5	H24A—C24A—H24B	107.9
H13D—C13B—H13E	109.5	C25B—C24B—C23B	112.00 (12)
N1B—C13B—H13F	109.5	C25B—C24B—H24C	109.2
H13D—C13B—H13F	109.5	C23B—C24B—H24C	109.2
H13E—C13B—H13F	109.5	C25B—C24B—H24D	109.2
C19A—C14A—C15A	117.75 (11)	C23B—C24B—H24D	109.2
C19A—C14A—C2A	120.70 (10)	H24C—C24B—H24D	107.9
C15A—C14A—C2A	121.55 (11)	N20A—C25A—C24A	113.15 (12)
C19B—C14B—C15B	117.37 (11)	N20A—C25A—H25A	108.9
C19B—C14B—C2B	121.12 (11)	C24A—C25A—H25A	108.9
C15B—C14B—C2B	121.49 (11)	N20A—C25A—H25B	108.9
C14A—C15A—C16A	121.29 (12)	C24A—C25A—H25B	108.9
C14A—C15A—H15A	119.4	H25A—C25A—H25B	107.8
C16A—C15A—H15A	119.4	N20B—C25B—C24B	112.74 (11)
C14B—C15B—C16B	121.38 (12)	N20B—C25B—H25C	109.0
C14B—C15B—H15B	119.3	C24B—C25B—H25C	109.0
C16B—C15B—H15B	119.3	N20B—C25B—H25D	109.0
C15A—C16A—C17A	121.14 (11)	C24B—C25B—H25D	109.0
C15A—C16A—H16A	119.4	H25C—C25B—H25D	107.8
			
C9A—N1A—C2A—C3A	−2.05 (18)	C8B—C9B—C10B—C4B	178.16 (12)
C13A—N1A—C2A—C3A	176.69 (11)	O12B—C4B—C10B—C5B	0.8 (2)
C9A—N1A—C2A—C14A	177.88 (11)	C3B—C4B—C10B—C5B	179.28 (12)
C13A—N1A—C2A—C14A	−3.38 (17)	O12B—C4B—C10B—C9B	−179.02 (12)
C9B—N1B—C2B—C3B	−2.25 (18)	C3B—C4B—C10B—C9B	−0.57 (17)
C13B—N1B—C2B—C3B	177.70 (12)	C3A—C2A—C14A—C19A	−68.97 (17)
C9B—N1B—C2B—C14B	178.07 (11)	N1A—C2A—C14A—C19A	111.11 (14)
C13B—N1B—C2B—C14B	−1.98 (17)	C3A—C2A—C14A—C15A	110.36 (14)
N1A—C2A—C3A—O11A	179.44 (11)	N1A—C2A—C14A—C15A	−69.56 (16)
C14A—C2A—C3A—O11A	−0.49 (17)	C3B—C2B—C14B—C19B	−85.44 (16)
N1A—C2A—C3A—C4A	1.92 (18)	N1B—C2B—C14B—C19B	94.23 (15)
C14A—C2A—C3A—C4A	−178.00 (11)	C3B—C2B—C14B—C15B	96.39 (15)
N1B—C2B—C3B—O11B	178.60 (11)	N1B—C2B—C14B—C15B	−83.93 (15)
C14B—C2B—C3B—O11B	−1.73 (18)	C19A—C14A—C15A—C16A	−1.90 (19)
N1B—C2B—C3B—C4B	−0.32 (19)	C2A—C14A—C15A—C16A	178.75 (11)
C14B—C2B—C3B—C4B	179.35 (11)	C19B—C14B—C15B—C16B	0.13 (19)
O11A—C3A—C4A—O12A	0.99 (18)	C2B—C14B—C15B—C16B	178.36 (12)
C2A—C3A—C4A—O12A	178.50 (11)	C14A—C15A—C16A—C17A	0.34 (19)
O11A—C3A—C4A—C10A	−177.63 (10)	C14B—C15B—C16B—C17B	0.38 (19)
C2A—C3A—C4A—C10A	−0.12 (17)	C15A—C16A—C17A—C18A	1.11 (19)
O11B—C3B—C4B—O12B	1.21 (18)	C15A—C16A—C17A—N20A	179.44 (12)
C2B—C3B—C4B—O12B	−179.86 (12)	C15B—C16B—C17B—C18B	−1.44 (19)
O11B—C3B—C4B—C10B	−177.27 (10)	C15B—C16B—C17B—N20B	176.69 (11)
C2B—C3B—C4B—C10B	1.66 (18)	C16A—C17A—C18A—C19A	−1.0 (2)
C10A—C5A—C6A—C7A	0.9 (2)	N20A—C17A—C18A—C19A	−179.35 (12)
C10B—C5B—C6B—C7B	0.6 (2)	C16B—C17B—C18B—C19B	2.1 (2)
C5A—C6A—C7A—C8A	0.9 (2)	N20B—C17B—C18B—C19B	−176.11 (12)
C5B—C6B—C7B—C8B	−0.8 (2)	C15A—C14A—C19A—C18A	2.0 (2)
C6A—C7A—C8A—C9A	−1.6 (2)	C2A—C14A—C19A—C18A	−178.62 (13)
C6B—C7B—C8B—C9B	−0.2 (2)	C17A—C18A—C19A—C14A	−0.6 (2)
C2A—N1A—C9A—C10A	0.34 (17)	C15B—C14B—C19B—C18B	0.5 (2)
C13A—N1A—C9A—C10A	−178.43 (11)	C2B—C14B—C19B—C18B	−177.74 (13)
C2A—N1A—C9A—C8A	179.67 (11)	C17B—C18B—C19B—C14B	−1.6 (2)
C13A—N1A—C9A—C8A	0.89 (17)	C16A—C17A—N20A—C25A	140.73 (13)
C7A—C8A—C9A—N1A	−178.84 (12)	C18A—C17A—N20A—C25A	−41.00 (17)
C7A—C8A—C9A—C10A	0.48 (19)	C16A—C17A—N20A—C21A	6.07 (18)
C2B—N1B—C9B—C10B	3.28 (17)	C18A—C17A—N20A—C21A	−175.65 (13)
C13B—N1B—C9B—C10B	−176.67 (12)	C16B—C17B—N20B—C21B	9.31 (17)
C2B—N1B—C9B—C8B	−176.71 (11)	C18B—C17B—N20B—C21B	−172.62 (12)
C13B—N1B—C9B—C8B	3.34 (18)	C16B—C17B—N20B—C25B	148.90 (13)
C7B—C8B—C9B—N1B	−178.50 (12)	C18B—C17B—N20B—C25B	−33.03 (17)
C7B—C8B—C9B—C10B	1.51 (19)	C17A—N20A—C21A—C22A	−176.03 (12)
N1A—C9A—C10A—C5A	−179.41 (11)	C25A—N20A—C21A—C22A	48.73 (17)
C8A—C9A—C10A—C5A	1.25 (18)	C17B—N20B—C21B—C22B	−175.92 (11)
N1A—C9A—C10A—C4A	1.49 (18)	C25B—N20B—C21B—C22B	44.34 (17)
C8A—C9A—C10A—C4A	−177.84 (11)	N20A—C21A—C22A—C23A	−53.71 (18)
C6A—C5A—C10A—C9A	−1.97 (19)	N20B—C21B—C22B—C23B	−50.97 (17)
C6A—C5A—C10A—C4A	177.14 (12)	C21A—C22A—C23A—C24A	56.61 (17)
O12A—C4A—C10A—C9A	179.83 (11)	C21B—C22B—C23B—C24B	57.25 (15)
C3A—C4A—C10A—C9A	−1.57 (17)	C22A—C23A—C24A—C25A	−56.29 (18)
O12A—C4A—C10A—C5A	0.74 (18)	C22B—C23B—C24B—C25B	−58.65 (16)
C3A—C4A—C10A—C5A	179.34 (11)	C17A—N20A—C25A—C24A	176.14 (12)
C6B—C5B—C10B—C9B	0.6 (2)	C21A—N20A—C25A—C24A	−48.48 (18)
C6B—C5B—C10B—C4B	−179.21 (13)	C23A—C24A—C25A—N20A	53.05 (19)
N1B—C9B—C10B—C5B	178.31 (12)	C17B—N20B—C25B—C24B	175.63 (12)
C8B—C9B—C10B—C5B	−1.70 (18)	C21B—N20B—C25B—C24B	−44.96 (17)
N1B—C9B—C10B—C4B	−1.84 (18)	C23B—C24B—C25B—N20B	52.75 (17)

### Hydrogen-bond geometry (Å, °)

**Table:**

*D*—H···*A*	*D*—H	H···*A*	*D*···*A*	*D*—H···*A*
O11A—H11A···O12A	0.84	2.32	2.760 (2)	113
O11A—H11A···O12B	0.84	1.95	2.723 (2)	153
O11B—H11B···O12A	0.84	2.01	2.791 (2)	154
O11B—H11B···O12B	0.84	2.30	2.741 (2)	113
C24B—H24D···O12A^i^	0.99	2.52	3.475 (2)	162

Symmetry codes:  (i) −*x*+1, −*y*+1, −*z*+1.
